# Selection Bias in Reporting of Median Waiting Times in Organ Transplantation

**DOI:** 10.1001/jamanetworkopen.2024.32415

**Published:** 2024-09-10

**Authors:** Simon Schwab, Andreas Elmer, Daniel Sidler, Lisa Straumann, Ueli Stürzinger, Franz Immer

**Affiliations:** 1 Swisstransplant, Bern, Switzerland; 2 Epidemiology, Biostatistics and Prevention Institute and Center for Reproducible Science, University of Zurich, Zurich, Switzerland; 3 Department of Nephrology and Hypertension, Inselspital, Bern University Hospital, University of Bern, Bern, Switzerland; 4 Swiss Transplant Cohort Study Patient Advisory Board, University Hospital Basel, Basel, Switzerland

## Abstract

**Importance:**

Median organ waiting times published by transplant organizations may be biased when not appropriately accounting for censoring, death, and competing events. This can lead to overly optimistic waiting times for all transplant programs and, consequently, may deceive patients on the waiting list, transplant physicians, and health care policymakers.

**Objective:**

To apply competing-risk multistate models to calculate probabilities for transplantation and adverse outcomes on the Swiss national transplant waiting list.

**Design, Setting, and Participants:**

The WAIT (Waitlist Analysis in Transplantation) study was a retrospective cohort study of all transplant candidates in Switzerland listed from January 1, 2018, or later and observed until December 31, 2023. Transplant candidates were listed in 1 of the 6 transplant centers (Basel, Bern, Geneva, Lausanne, St Gallen, and Zurich) for heart, liver, lungs, kidney, or pancreas and/or islet transplant. A total of 4352 candidates were listed during the study period, of whom 709 (16.3%) were excluded due to living-donor transplant (691 in the kidney program and 18 in the liver program).

**Exposure:**

Waiting for organ transplant.

**Main Outcomes and Measures:**

Time to transplantation, death, or delisting. Competing-risk multistate models were used to analyze time-to-event data from the national organ waiting list with the Aalen-Johansen estimator to compute probabilities for both transplant and adverse outcomes. Results were compared with the sample median among only those undergoing transplant and the Kaplan-Meier method with censoring of competing events.

**Results:**

Data from 3643 transplant candidates (2428 [66.6%] male; median age, 56 [range, 0-79] years) were included in the analysis. The median time to transplantation (MTT) was 0.91 (95% CI, 0.83-1.07) years for heart, 3.10 (95% CI, 2.57-3.77) years for kidney, 1.32 (95% CI, 0.76-1.55) years for liver, 0.80 (95% CI, 0.37-1.12) years for lung, and 1.62 (95% CI, 0.91-2.17) years for pancreas and/or islet programs. Alternative estimation methods introduced bias to varying degrees: the sample median among only persons undergoing transplantation underestimated the waiting time by 38% to 61% and the Kaplan-Meier method by 2% to 12% compared with the MTT.

**Conclusions and Relevance:**

In this cohort study of transplant candidates in Switzerland, the MTT, the duration at which the transplant probability is 0.50, was used as a measure of average waiting time. Suboptimal methods led to biased and overly optimistic waiting time estimations; thus, applying appropriate competing-risk methods to address censoring and competing events is crucial.

## Key Points

**Question:**

What is the best way to estimate how long candidates wait to receive an organ transplant in Switzerland?

**Findings:**

In a national cohort study of 3643 participants, the median time to transplant was 0.9 years for heart, 3.1 years for kidney, 1.3 years for liver, 0.8 years for lungs, and 1.6 years for pancreas and/or islet. Competing-risk multistate models were more appropriate than median waiting time among recipients, which underestimated waiting times and had selection bias; the Kaplan-Meier estimator also slightly underestimated waiting times.

**Meaning:**

These findings suggest that transplant organizations should adopt appropriate competing-risk methods to address censoring and competing events when reporting waiting times in organ transplantation.

## Teaser

This cohort study applies a competing-risk multistate model to compute probabilities for both transplant and adverse outcomes among individuals on the Swiss national transplant waiting list.

## Introduction

Transplant candidates seek information from transplant organizations and centers, asking questions like, “What is the probability of receiving an organ?” and “How long is the expected waiting time?” While seemingly straightforward, these questions are not easily addressed. The complexity arises on 3 fronts. First, not all candidates on the waiting list will experience an outcome (censoring). Second, different biases may emerge in study design and analysis. Last, the competing risks must be addressed appropriately.^1^

A rather simplistic approach is to calculate the median across the waiting times among only transplant recipients. Typically, the distribution of recipient waiting times is right skewed; thus, the median is commonly used to measure central tendency. However, selection bias occurs when patients in a study or an analysis differ systematically from the population of interest. Recipients are a subpopulation from the population of transplant candidates on the waiting list. This questions the generalizability of the median waiting time among recipients to everyone on the waiting list. It is not acknowledged that a person on the waiting list may die, or no event may be observed (censoring).

It has been recognized that relying on the median waiting time among recipients could be misleading. Two studies that primarily considered kidney recipients^2,3^ recommended censoring competing events and using the Kaplan-Meier estimator or a Cox proportional hazards model. However, the Kaplan-Meier approach may result in an upward bias in the cumulative incidence of transplantation and should be reconsidered in favor of competing-risk methods.^4,5,6,7,8^ The Kaplan-Meier method assumes that censoring is independent or noninformative, which means that individuals who remain under follow-up have the same risk for the occurrence of an event as those who have been censored. However, this assumption may be violated when competing events are censored because it is unreasonable to assume that individuals who died on the waiting list (and thus were treated as censored) can be represented by those who remained on the waiting list and had no event.^1,7^

Alternative strategies for analyzing the organ waiting list are widely available^9,10^ but not yet established in the Swiss transplant setting. Hence, this study applied a competing risk multistate model using the Aalen-Johansen estimator to compute probabilities for both transplantation and adverse outcomes within the Swiss national transplant waiting list.

## Methods

### Study Design

The WAIT (Waitlist Analysis in Transplantation) study was a national retrospective cohort. The study encompassed all individuals listed on the national organ waiting list on January 1, 2018, or later and observed until December 31, 2023 (end of study date). Our cohort study does not fall under the Human Research Act; legal regulation in Switzerland follows an internationally applied distinction between research subject to approval and quality assurance that is not subject to approval. Therefore, the Ethics Committee of the Canton of Bern determined the study to be exempt from review and the requirement to obtain informed consent. The present study meets the criteria for a quality assurance project in terms of reporting official national statistics of transplant activity and outcomes on the waiting list. According to the Swiss Organ Allocation Ordinance, the national organ allocation office (Swisstransplant) compiles statistics on the donation, allocation, and transplant of organs (Article 34d lit. c).^11^ We followed the Strengthening the Reporting of Observational Studies in Epidemiology (STROBE) reporting guidelines.

### Setting

On behalf of the Federal Office of Public Health, Swisstransplant, the Swiss National Foundation for Organ Donation and Transplantation, is responsible for allocating organs to recipients in accordance with the law and maintains the national waiting list. In 2023, 584 organs from deceased donors were transplanted, with 1391 transplant candidates remaining on the waiting list as of the end of the same year. Switzerland has 6 transplant centers at the University Hospitals of Basel, Bern, Geneva, Lausanne, and Zurich, as well as at the Cantonal Hospital of St Gallen. The transplant centers decide who is included on the national organ waiting list and who is suitable for a transplant based on the organ quality and the recipient’s medical history.

### Participants

A total of 4352 candidates were listed during the study period from January 1, 2018, to December 31, 2023. All candidates were followed up from the listing date to the end of the study period. Persons with a planned living donation at the date of listing or persons who received a living-donor organ were excluded from the analysis; these consisted of 691 (15.9%) in the kidney transplant program and 18 (0.4%) in the liver transplant program. The waiting times of living-donor transplant recipients are independent of the availability of deceased donor organs.

The resulting analysis dataset involved 3643 transplant candidates, of whom 109 were listed for multiorgan transplantation. The analyses were conducted separately for each organ transplant program, ensuring no dependencies in the analyses due to multiorgan transplantation. There was no loss to follow-up or missing data. No race or ethnicity variables were collected.

We used an incidence cohort and did not include recipients listed before 2018, even though they may have received a transplant during the observation period. Including these individuals, also known as the prevalent cohort, would introduce various biases.^12^ For example, there can be immortal time bias because some persons have immortal time until January 1, 2018, and also survivorship bias because others would be excluded due to left truncation.

### Data Source and Outcome

The data source was the Swiss Organ Allocation System, which is used by Swisstransplant for national organ allocation and also contains the national waiting list for each organ. The outcome was the time from listing to transplantation, death, or delisting, whatever occurred first.

### Multistate Models

**Figure 1.**
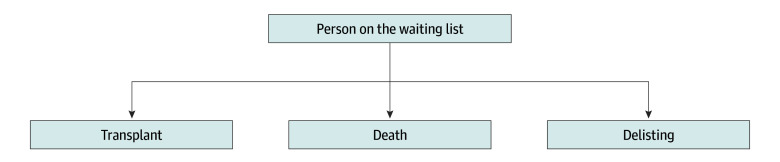
Multistate Model for the Organ Waiting List With Transplantation, Death, and Delisting as Competing Events

In a multistate model, persons can transition from one state to another.^9,10^ For example, on the transplant waiting list, persons can transition from the waiting list to 3 states: transplant, death, or delisting (Figure 1). In survival analysis, these are known as competing risks, that is, when other events (competing risks) preclude the occurrence of the primary event (transplant). For example, death or delisting will exclude transplantation. Likewise, a transplant recipient cannot die anymore on the waiting list. Only transitions from the initial state to one of the competing-risk states are allowed (absorbing states). Delisting is a third state that may occur, for example, when a person is considered too sick or too old for transplantation.^13^

For the estimation of the transition probabilities, the Aalen-Johansen estimator can be used; it is an extension of the Kaplan-Meier estimator for a Markov process with a finite number of states. The resulting cumulative incidence curves for each state are very natural to interpret as the probability that an event occurs in a given period. The Aalen-Johansen estimator has the elegant property that the probability of transplantation until time *t*, plus the probability of death until *t*, plus the probability of delisting until *t*, plus the probability of still waiting until *t*, is exactly 1.

### Statistical Analysis

For descriptive statistics, we used the median and IQR for continuous variables, and frequencies and percentages for dichotomous and categorical variables. The median follow-up time was estimated by reverse Kaplan-Meier with a reversed status indicator.^2^ In this approach, the events on the waiting lists become censored observations, and the censored observations become events to assess the median length of follow-up.

We fitted a multistate model using survfit from the survival package, version 3.7-0,^14,15^ using the Aalen-Johansen estimator for multistate models. All analyses were performed using R, version 4.4.1 (R Project for Statistical Computing).^16^

## Results

We analyzed time-to-event data from 3643 transplant candidates (1215 [33.4%] female and 2428 [66.6%] male; median age, 56 [range, 0-79] years); characteristics of the cohorts per transplant program are shown in Table 1. All transplant candidates were listed between January 1, 2018, and December 31, 2023. A total of 109 candidates (3.0%) were listed for multiorgan transplantation, most commonly kidney and pancreas (72 [66.1%]) and liver and kidney (26 [23.9%]). The median follow-up time across all programs was 2.93 (IQR, 2.46-3.63) years.

**Table 1.** Characteristics of the Swiss Transplant Candidates in 5 Major Transplant Programs

Characteristic	Patients, No. %^a^				
	Heart (n = 306)	Kidney (n = 1662)	Liver (n = 1321)	Lungs (n = 345)	Pancreas and/or islet (n = 118)
Age, median (IQR), y	51 (34-58)	56 (45-64)	58 (48-64)	57 (48-61)	46 (37-53)
Sex					
Female	80 (26.1)	560 (33.7)	413 (31.3)	151 (43.8)	57 (48.3)
Male	226 (73.9)	1102 (66.3)	908 (68.7)	194 (56.2)	61 (51.7)
Multiple transplants	7 (2.3)	106 (6.4)	29 (2.2)	1 (0.3)	76 (64.4)
Urgent listings	28 (9.2)	1 (0.1)	113 (8.6)	17 (4.9)	0
Blood type					
A	134 (43.8)	709 (42.7)	588 (44.5)	157 (45.5)	47 (39.8)
AB	15 (4.9)	57 (3.4)	59 (4.5)	20 (5.8)	4 (3.4)
B	47 (15.4)	194 (11.7)	156 (11.8)	37 (10.7)	11 (9.3)
O	110 (35.9)	702 (42.2)	518 (39.2)	131 (38.0)	56 (47.5)

^a^ Unless otherwise indicated, data are expressed as No. (%) of participants. A total of 109 of 3643 participants were on the organ transplant waiting list for multiple organs.

Across all transplant programs, the following transitions were observed: 2035 transplants, 327 deaths, and 204 delistings. At the end of the study, 1186 observations were censored. These observations surpass the total number of transplant candidates due to the 109 multiorgan listings.

Probabilities of being in each state after 1, 2, 3, 4, and 5 years since listing are shown in Table 2. For example, the probability of receiving a heart transplant within the first year was 52%; the probability of still waiting, 36%; the probability of death, 8%; and the probability of delisting, 3%. In the kidney transplant program, the probability of receiving a transplant was 20% within the first year, 33% by the second year, 49% by the third year, 59% by the fourth year, and 71% by the fifth year.

**Table 2.** Probabilities of the States for Different Waiting Durations Since the Time of Listing for the 5 Transplant Programs

Transplant program	State probability				
	After 1 y	After 2 y	After 3 y	After 4 y	After 5 y
Heart					
Received transplant	0.52	0.64	0.70	0.77	0.78
Still waiting	0.36	0.19	0.12	0.03	0.02
Death	0.08	0.12	0.12	0.13	0.13
Delisting	0.03	0.05	0.06	0.07	0.07
Kidney					
Received transplant	0.20	0.33	0.49	0.59	0.71
Still waiting	0.77	0.61	0.42	0.30	0.15
Death	0.01	0.03	0.05	0.06	0.07
Delisting	0.01	0.03	0.04	0.05	0.07
Liver					
Received transplant	0.40	0.63	0.67	0.68	0.69
Still waiting	0.44	0.16	0.08	0.04	0.02
Death	0.12	0.14	0.16	0.17	0.17
Delisting	0.04	0.07	0.10	0.11	0.12
Lungs					
Received transplant	0.55	0.77	0.83	0.85	NA
Still waiting	0.37	0.12	0.04	0.01	NA
Death	0.07	0.08	0.09	0.10	NA
Delisting	0.02	0.03	0.04	0.04	NA
Pancreas and/or islet					
Received transplant	0.42	0.58	0.70	0.72	0.75
Still waiting	0.57	0.36	0.23	0.21	0.12
Death	0.01	0.03	0.03	0.03	0.06
Delisting	0.00	0.03	0.04	0.04	0.08

Abbreviation: NA, not applicable.

**Figure 2.**
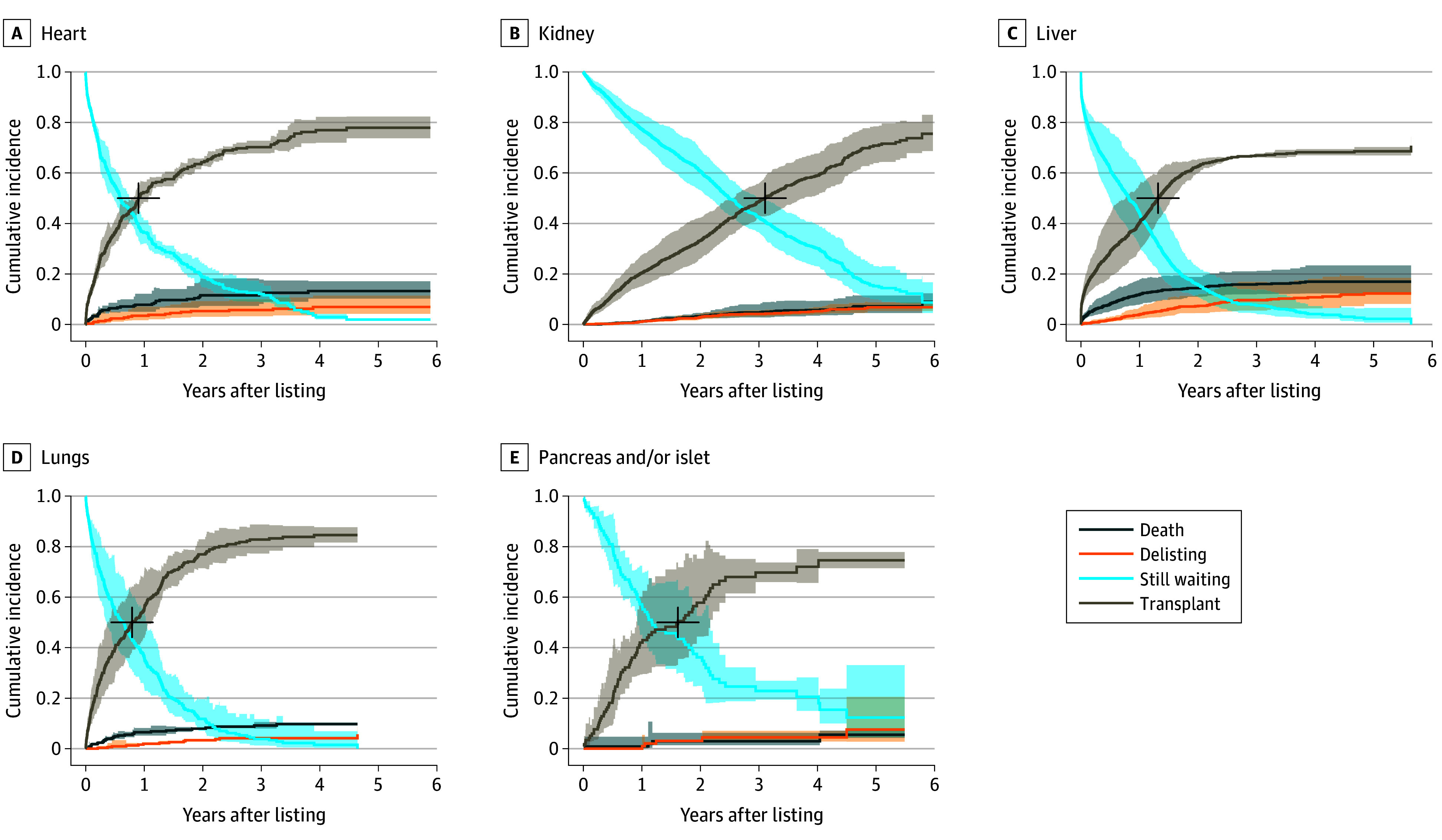
Cumulative Incidence Curves for All States per Transplant Program Median time to transplantation is defined as the duration corresponding to the cumulative incidence of 0.50 (cross mark). Shading indicates 95% pointwise confidence bands.

We show the cumulative incidence curves for all transplant programs for the 3 possible outcomes (transplantation, death, and delisting) in Figure 2. The probability of transplantation goes beyond 50% within the first 2 years after listing in all the transplant programs except in kidney transplantation. The cumulative incidence of kidney transplantation follows an almost linear trajectory over the years, and even after 5 years, there remains a 15% probability of still being on the waiting list.

After 4 years, the probabilities of waiting for a heart, a liver, or lungs decreased to nearly zero. Among the various programs, the liver program presented the highest probability of death within the first year (12%), followed by the heart (8%), lungs (7%), kidney (1%), and pancreas and/or islet (1%) programs. Delisting was the least frequent outcome, predominantly occurring on the liver and heart waiting lists, with probabilities of 11% and 7%, respectively, by 4 years.

### Median Time to Transplantation

In survival analysis of clinical trials, it is common to report the median survival time to evaluate the efficacy of a novel treatment. It is the length of time corresponding to a probability of 0.50 of surviving this time point. Applying this concept to the waiting list, the median time to transplantation (MTT) represents the duration since listing that aligns with a 0.50 probability of undergoing transplantation. To illustrate this, we highlighted the MTT (cross mark) in Figure 2 and provided the estimates in Table 3. As it may be important to communicate risks to patients, the probability of death and delisting during MTT was also amended. The MTT was 0.91 (95% CI, 0.83-1.07) years for heart, 3.10 (95% CI, 2.57-3.77) years for kidney, 1.32 (95% CI, 0.76-1.55) years for liver, 0.80 (95% CI, 0.37-1.12) years for lung, and 1.62 (95% CI, 0.91-2.17) years for pancreas and/or islet programs.

**Table 3.** Direct Comparison of Different Approaches to Calculate Waiting Time Estimates^a^

Transplant program	Sample median waiting time among transplant recipients, y (n = 1976)^b^	Kaplan-Meier median waiting time (95% CI), y (n = 3643)^c^	Competing risk multistate model with Aalen-Johansen (n = 3643)		
			MTT (95% CI), y	Probability of death during MTT	Probability of delisting during MTT
Heart	0.49	0.85 (0.59-0.95)	0.91 (0.83-1.07)	0.08	0.03
Kidney	1.34	2.93 (2.46-3.63)	3.10 (2.57-3.77)	0.05	0.04
Liver	0.52	1.16 (0.77-1.50)	1.32 (0.76-1.55)	0.13	0.05
Lungs	0.50	0.76 (0.45-1.14)	0.80 (0.37-1.12)	0.06	0.01
Pancreas and/or islet	0.70	1.59 (0.93-2.21)	1.62 (0.91-2.17)	0.03	0.03

Abbreviation: MTT, median time to transplant.

^a^ Populations included were all added to the waiting list between January 1, 2018, and December 31, 2023.

^b^ Ignores competing events and censoring.

^c^ Competing events are censored.

We compared the MTT to the simple median among recipients in Table 3. For example, the median across all kidney recipients was 1.34 years; however, the MTT was 3.10 (95% CI, 2.57-3.77) years. The median transplant times among recipients showed a downward bias, being 46% too short for the heart, 57% for the kidney, 61% for the liver, 38% for the lungs, and 57% for the pancreas and/or islet programs.

We also compared the MTT with the Kaplan-Meier estimate; the Kaplan-Meier estimate for median waiting time also had a downward bias. This was expected, as the Kaplan-Meier estimate had an upward bias for the event incidence when competing events were censored. The relative biases of the median waiting time using Kaplan-Meier were 7% too short for the heart, 5% for the kidney, 12% for the liver, 6% for the lungs, and 2% for the pancreas and/or islet transplant programs.

## Discussion

In this cohort study, we calculated the MTT with an established method from competing-risk literature in the Swiss population of transplant candidates listed between January 1, 2018, and December 31, 2023. We presented the MTT, the waiting time at which the probability of transplantation is 50%. In other words, and more informally speaking, the MTT is the time by which 50 of 100 persons on the waiting list are expected to receive a transplant; thus, it can indeed be seen as an average waiting time.

We found that simply computing the median of waiting times among recipients yielded highly downward-biased estimates, which can mislead patients, authorities, and health care professionals. Although the bias may be smaller when considering all recipients during the observation period (prevalent cohort) instead of the newly listed ones (incidence cohort), the underlying approach still has conceptual flaws.

It is widely acknowledged that Kaplan-Meier provides biased results in the presence of competing risks.^4,8,17^ Our study has quantified this bias in the Swiss population of transplant candidates, revealing a relative bias compared with MTT between 5% and 12%, depending on the particular transplant program. The liver transplant program was particularly susceptible to this bias because the competing risks of death and delisting occurred more often in this program than in others. When using Kaplan-Meier and censoring death and delisting, the assumption for these censored observations was that transplantation can occur in the future. However, in reality, the probability of transplantation was 0 for these individuals.

The bias, whether using the median among recipients or Kaplan-Meier estimate, always operated in the direction of underestimating the waiting time, giving too much optimism, and raising ethical considerations. It has been suggested that no evidence for bias was found when using a prevalent cohort instead of an incidence cohort.^2^ However, this may not apply to other settings, and this absence of evidence should not be mistaken for evidence of absence. Therefore, we suggest considering competing risk methods over Kaplan-Meier, but the choice also depends on the research question being addressed. Using an incidence cohort in the design and analysis safeguards against selection bias and immortal time bias, eliminating the necessity for additional statistical procedures to address these issues.

The Aalen-Johansen and the Kaplan-Meier methods estimate different quantities, often referred to as different estimands. Specifically, the Aalen-Johansen method estimates the actual probability of the event of interest in the presence of competing events, reflecting a more realistic scenario when multiple types of events are possible. This information is useful for counseling a new patient placed on the waiting list who wants to know the probability of transplantation under the assumption that other events (death and delisting) may also occur. However, there are scenarios where a competing-risk cumulative incidence may not be of interest. For example, if the interest is in the risk of death if a patient would never undergo transplantation (ie, a hypothetical scenario if transplantation was banned from medical practice), then Kaplan-Meier estimates with censoring transplantation may be more appropriate.^18^ This information may be useful to patients who are still considering whether or not to undergo transplantation. Thus, the specific research question should guide the decision to apply Aalen-Johansen or censor competing events.

We excluded individuals with a planned living donation at the time of listing or individuals who received a living-donor organ. Candidates who were initially waiting for a deceased-donor transplant and later switched to pursuing living-donor transplant but had not yet received a transplant were included in the analysis. Others have suggested censoring for living-donor transplant.^19,20^

Another issue arises from individuals on the waiting list who are inactive, excluding them from receiving an organ offer and transplant (eg, acute infection, psychological reasons, cardiac reasons). We decided not to account for this inactive status because the aim of the study was to provide an accurate estimate of a prospective waiting time (on the population level). In other words, we wanted to predict rather than explain.^21^ Therefore, persons in inactive status should not be excluded, nor should the result be adjusted for it (eg, by subtracting inactive time from the total waiting time). Doing so may result in too optimistic estimates for a hypothetical world without inactive status.

Communicating with patients on the organ waiting list about their prognosis, likelihood of transplantation, and potential adverse outcomes can be challenging. First, it is certainly an emotional subject with large uncertainties. Second, the probabilities are based on groups of people and may be difficult to relate to an individual. Third, it may be hard to balance statistical accuracy and simple terms. Last, the patient’s psychological state must be considered when communicating risks. In the future, individualized prognosis through regression-based prediction models may be used to offer more tailored estimates of waiting times.^3,22^

### Limitations

This study has some limitations. First, the results only apply to the Swiss population of transplant candidates and cannot be generalized to transplant programs in other countries. Still, we hope to provide convincing arguments so that transplant organizations do not report average waiting times without considering death and censored observations appropriately. Second, the results do not apply to candidates listed before January 1, 2018. Third, MTT obviously cannot be calculated for transplant programs in which the 0.50 probability is not reached within a reasonable timeframe. Then, other quantiles, including lower ones like the 25th percentile, must be computed, or one can report on the probability of transplantation after 1 year, 2 years, and so on.

## Conclusions

Findings of this cohort study suggest that understanding the waiting list as a competing-risk multistate model can appropriately inform patients and clinicians about average expected waiting times and support the monitoring of national transplant programs. Competing risk methods should be preferred over the simple median among recipients or Kaplan-Meier to avoid bias and overoptimistic estimates.

## Notes

Supplement.
Data Sharing Statement
