# Error in Figure 2

**DOI:** 10.1001/jamanetworkopen.2024.42745

**Published:** 2024-10-07

In the Original Investigation titled “Selection Bias in Reporting of Median Waiting Times in Organ Transplantation,”^1^ published September 10, 2024, there was an error in Figure 2. In each panel, the x-axis given as “Years after transplant” should have been “Years after listing.” This article has been corrected.^1^
