# Supplementary material for: Selection Bias in Reporting of Median Waiting Times in Organ Transplantation
Source: JAMA Netw Open. 2024 Sep 10;7(9):e2432415. doi: 10.1001/jamanetworkopen.2024.32415 (PMC11388028; doi:10.1001/jamanetworkopen.2024.32415)
Supplement: Supplement. — Data Sharing Statement [file jamanetwopen-e2432415-s001.pdf]

**Data Sharing Statement**

**Data**
**Data available:** No

**Additional Information**
**Explanation for why data not available:** The data supporting this study's findings are available from the Federal Office of Public Health (FOPH), Switzerland. Restrictions apply to the availability of these data.
